# Open-Label Placebo Treatment: Outcome Expectations and General Acceptance in the Lay Population

**DOI:** 10.1007/s12529-020-09933-1

**Published:** 2020-10-22

**Authors:** Julia W. Haas, Winfried Rief, Bettina K. Doering

**Affiliations:** 1 Department of Clinical Psychology and Psychotherapy, Philipps-University of Marburg, Marburg, Germany; 2 Department of Clinical and Biological Psychology, Catholic University of Eichstätt-Ingolstadt, Eichstätt, Germany

**Keywords:** Placebo, Online experiments, Vignette measure, Attitude measurement

## Abstract

**Background:**

Most physicians sometimes apply therapies without specific active ingredients. Although patients seem to judge such placebo treatments as acceptable under certain circumstances, deception is still an ethical problem. Open-label placebos (OLPs) might be a promising approach to solve this dilemma. This study compared general acceptance and outcome expectations of OLPs and deceptive placebos (DPs).

**Methods:**

In an experimental online study, 814 participants read a case vignette of a person with insomnia receiving a pill. The participants were then randomly allocated into two groups, where the second part of the vignette described the pill as either a deceptive placebo (DP group) or as an open-label placebo (OLP group). The Credibility/Expectancy Questionnaire (CEQ) assessed outcome expectations after the first (pre-assessment) and the second (post-assessment) parts of the vignette. Treatment acceptance was measured at post-assessment. Data from 798 participants were analyzed by a mixed multivariate analysis of variance (MANOVA), *t-*tests, and post-hoc mediation analyses.

**Results:**

The MANOVA revealed a significant group main effect and a significant time × group interaction effect. At post-assessment, outcome expectations were higher in the DP group than in the OLP group. Acceptance of the placebo treatment was also higher in the DP group than in the OLP group. Mediation analyses confirmed that higher acceptance in the DP group was mediated by higher expectations.

**Conclusions:**

When laypersons read about placebo treatment, their outcome expectations toward DPs were higher than toward OLPs. Surprisingly, the application of DPs was rated as more acceptable than OLPs. This result might be explained by indirect effects of treatment expectations.

**Electronic supplementary material:**

The online version of this article (10.1007/s12529-020-09933-1) contains supplementary material, which is available to authorized users.

## Introduction

According to systematic reviews, placebo application is quite common in clinical practice. Although results vary considerably between different studies and different countries, a substantial number of physicians have obviously applied placebos of some form at least once in their careers [1, 2]. This is not limited to pure placebos, meaning substances without any active agent, like sugar pills. Likewise, active therapies are, by definition, impure placebos when they are not supposed to affect the specific symptoms for which they are prescribed. Examples of impure placebos include antibiotics prescribed against viral infections, vitamin pills, or medication that is too low-dosed to cause pharmacological effects. Usually, patients are not informed, in these cases, that the therapies they receive have no specific effect on their complaints. Thus, applying impure placebos involves equal deception to pure placebos. While only a minority of physicians seem to apply pure placebos regularly, the majority (53–89%) use impure placebos, i.e., non-specific therapies, at least monthly [2]. Further, eliciting beneficial placebo effects was a frequently mentioned reason for applying impure placebos [1, 2].

A survey among patients and general practitioners in Switzerland indicates that patients tend to rate such placebo treatments as even more acceptable than physicians themselves [3]. Furthermore, in a study with US patients, only 21.9% stated that it was never acceptable for physicians to apply a placebo treatment, while the vast majority judged placebo treatments as acceptable as long as physicians were certain about the benefits and the lack of harm [4, 5]. Similarly, after receiving comprehensive information about beneficial placebo effects in depression, both healthy participants and patients with major depressive disorder in Israel expressed high rates of willingness to receive placebo treatments for future depression or general medical conditions. The majority of these participants did not even feel this would be deceitful or diminish their sense of autonomy [6]. However, deception remains an ethical problem. Different studies indicate that patients still regard honesty, transparency, and respect of their autonomy as crucial for medical treatments, including the use of placebos [4, 5, 7]. Furthermore, physicians seem to underestimate how many of their patients want to be explicitly informed before receiving non-specific therapies [3]. Open-label placebo (OLP) application seems to be a promising approach to reconcile patients’ and doctors’ agreement to placebo use, as well as the patients’ right for transparency.

OLPs are placebos that are prescribed honestly, i.e., by informing patients they receive a placebo without deception. While OLP application was first investigated in an uncontrolled pilot study by Park and Covi in 1965 [8], the first trial on OLP including a no-treatment control group was implemented by Kaptchuk and colleagues [9, 10]. The information they provided to patients, a practice which was often adopted in later OLP studies, included information about the positive effects of placebos on different symptoms, a brief explanation of classical conditioning as a potential placebo mechanism, and the indication that a positive attitude toward the intervention might be beneficial but not necessary. This implies that typical OLP applications are also combined with the verbal induction of positive outcome expectations. There is increasing evidence of positive clinical effects of OLPs on different medical conditions [11], e.g., irritable bowel syndrome [9], chronic pain [12, 13], allergic rhinitis [14], cancer fatigue [15, 16], and migraines [17]. These results indicate that especially those medical conditions, which cannot be attributed to clear and treatable causes and manifested in rather unspecific symptoms without dangerous consequences, seem to benefit from OLP treatment. For these conditions, OLPs seem to be a favorable alternative to deceptive placebos (DPs), including the aforementioned non-specific therapies. Given that laypeople seem to evaluate placebo application as unacceptable when they prioritize the deception over other issues [7], OLPs should be rated as more acceptable because they give patients the opportunity for an autonomous decision. This idea is supported by a telephone survey which indicated high acceptance of OLPs in laypeople; more than three-quarters of respondents thought it was acceptable for physicians to offer an OLP treatment for moderate, non-serious stomach pain [4]. Still, studies comparing the acceptance of DP and OLP treatments in the lay population, which would answer the question whether OLPs are rated as the more acceptable method of placebo application, are lacking.

Acceptance of placebo treatments is related not only to transparent information but also to participants’ outcome expectations. Qualitative data provides evidence that placebos are only rated as acceptable when they are expected to be effective [7]. With DPs, outcome expectations constitute an essential underlying mechanism [18] and the induction of positive expectations augments placebo effects [19, 20]. However, evidence on the role of expectations in OLP treatment is mixed. In allergic rhinitis, one trial suggests that a plausible rationale inducing positive expectations is necessary to facilitate OLP effects [21], while in another study, the success of OLP treatment did not depend on the rationale, i.e., the enhanced expectancy [22]. Furthermore, many patients who participated in OLP studies and benefitted from the intervention denied having positive expectations. Instead, they often reported that they had undergone multiple unsuccessful treatments before, and therefore, they felt both despair and a strong desire for relief [10, 23, 24]. This interaction of despair and desire for relief might be a more important mechanism of OLP effects than expectations. Additionally, many laypersons seem to believe that placebos need to be applied with deception to have an effect [7]. Therefore, it can be assumed that outcome expectations are lower toward OLPs than DPs in the lay population.

Keeping in mind that (a) DP application does occur quite often in routine healthcare and (b) patients seem to endorse such placebo treatments but also wish to be honestly informed, investigating the applicability of OLPs in medical practice is important. Laypersons’ acceptance of OLPs has been examined [4] but never compared with their acceptance of DPs. Expectations towards OLP have been investigated regarding their role as a potential OLP mechanism [21, 22], and regarding the placebo effect, they can evoke in this context as compared with DP treatment [25]. However, to the best of our knowledge, no study has compared both outcome expectations and treatment acceptance of DPs and OLPs systematically. Our study set out to assess these two aspects in one design and include a large sample of the lay population.

We implemented an online vignette study to investigate, experimentally, how general acceptance and outcome expectations toward DPs and OLPs differ in the lay population. We chose sleep problems as the medical condition of interest because it often cannot be attributed to clear and treatable causes. After all participants had read a case vignette about a person suffering from insomnia and receiving a pill to aid the sleep problems, they were randomized into two groups. Depending on their allocation, they were assigned to subsequently read a different treatment vignette. In the deceptive placebo group (DP group), the treatment vignette described the pill as a DP, but in the open-label placebo group (OLP group), it was described as an OLP. Outcome expectancy and rationale credibility were measured before and after the treatment vignette, and treatment acceptance was measured afterwards. It was hypothesized that outcome expectancy would be higher in participants who read the DP treatment vignette, whereas treatment acceptance would be higher in participants who read the OLP treatment vignette.

## Methods

### Participants and Study Procedure

Participants were recruited in September and October 2018 via mailing lists from the university, social media, and flyers in public buildings. The only inclusion criterion was a minimum age of 18 years. According to an a priori power analysis with G*Power 3.0.8 [26], 787 participants were required in order to detect a within-between interaction effect with small effect size (*f* = 0.10) using a multivariate analysis of variance with two groups and two measurement points, an *α* level of 0.05 and *β* of 0.80. To account for potential dropouts, the intended sample size was 800.

The online vignette study was approved by the local ethics committee of the university. It was implemented using SoSci Survey [27] and provided to participants at www.soscisurvey.de. Participation took approximately 15 min, and the survey could be accessed from any device with internet access. Before starting the survey, participants had to give informed consent actively by ticking a box. Demographical data was assessed, the Beliefs about Medicines Questionnaire [28] had to be completed, and participants were asked about their knowledge and attitude concerning both placebos and soporifics. To ensure a similar level of knowledge in all participants, a short definition of the placebo effect was given, which was adopted from Fässler and colleagues [3]. Afterwards, participants read a case vignette which instructed them to imagine suffering from insomnia.

**Case vignette (all participants)***: Imagine you have suffered from sleep problems for a couple of months. In the evenings you often lie awake for one or two hours. […] As a result, you feel tired and exhausted in the daytime. […] You make an appointment in a specialized sleep laboratory. […] The doctor offers you a medication against the sleeping disorder. […] “I recommend you try these pills. This is a good and effective remedy against sleeping disorders and only has a few side effects. […].”*

After this case vignette, participants had to complete the Credibility/Expectancy Questionnaire (CEQ) [29] and were then allocated randomly to one of two groups: the DP group or the OLP group. Depending on their allocation, they received a different treatment vignette. Both treatment vignettes had approximately the same word count and described a placebo intervention, either with deception or openly.

**Treatment vignette DP group**:* The doctor does not tell you that the pills are in fact placebos without an active agent. You do not even consider the pills to be a fake medication. […] You lie down to sleep in the belief that you have taken a sleeping pill with active agent.*

**Treatment vignette OLP group:*** The doctor explains, “These pills are placebos without an active agent. Scientific research has shown that placebos can have a large positive effect on sleeping disorders and can improve symptoms.[…] The body can respond automatically to the intake although you know that it is placebo.[…].”*

**Fig. 1:**
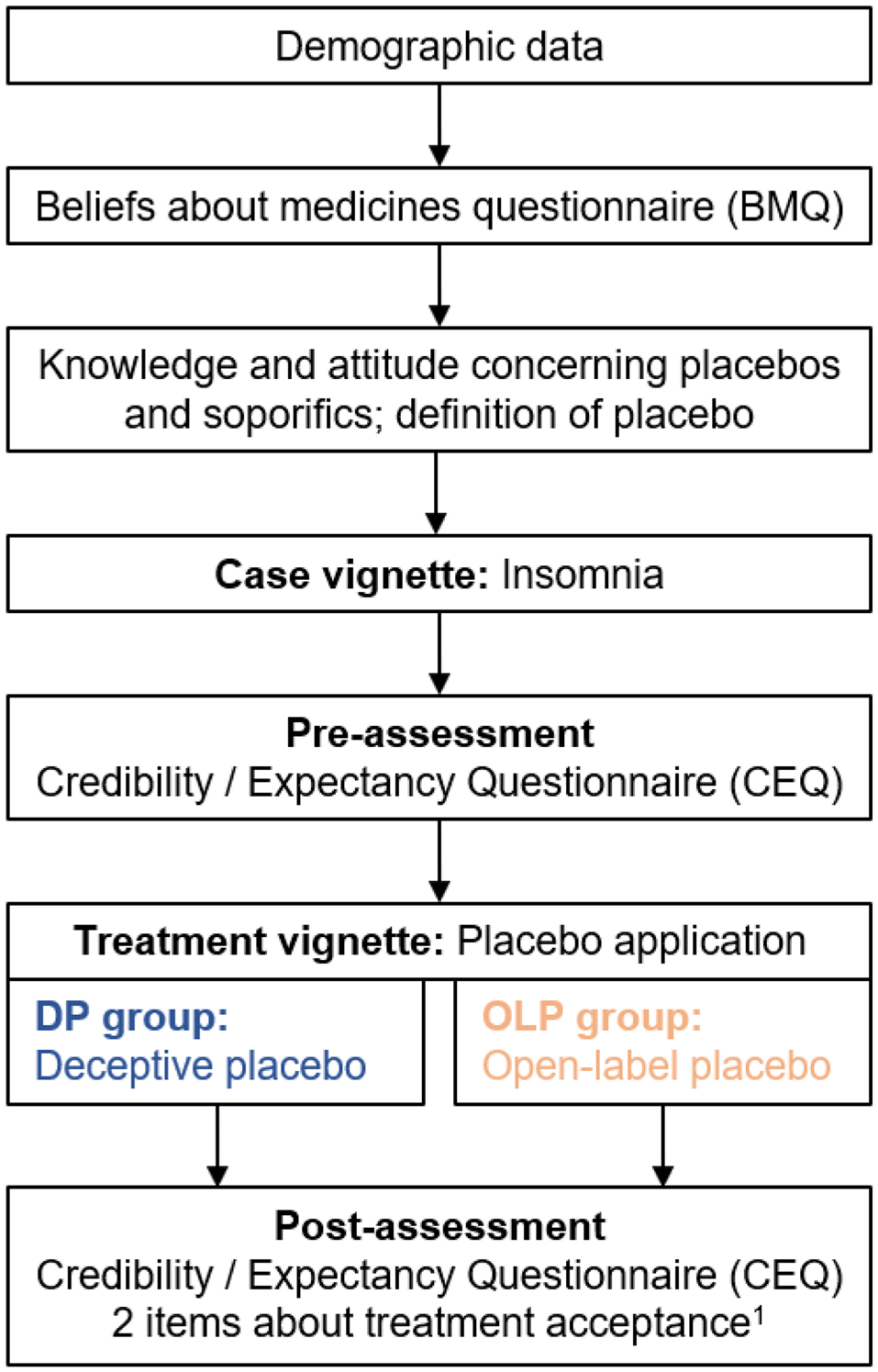
Procedure of the online study. The vignettes can be read in full length in the supplementary material

Participants then completed the CEQ for the second time. Further, they answered two items concerning their treatment acceptance, which were adopted from Ortiz, Hull, and Colloca [5]. The procedure of the study is shown in Fig. 1. The vignettes can be read in full length in the supplementary material.

### Measures

#### Credibility/Expectancy Questionnaire

To investigate the participants’ expectancy toward the described placebo interventions, the CEQ [29] was used. This questionnaire assesses the credibility of a treatment’s rationale, which is a rather cognitively based factor, and the expectancy toward the treatment’s effectiveness, which is more affectively based. Both factors contain three items each, which are all answered on numeric rating scales, ranging either from 1 (“not at all”) to 9 (“very much”) or from 0 to 100%. For the evaluation, all scores need to be standardized before being summarized to the two factors. Both factors are assumed to have good internal consistency (Cronbach’s *α* = .79–.90) and good test-retest reliability (credibility .75; expectancy .82) [29]. Content validity and the two-factor structure of the CEQ have been confirmed [30, 31].

#### Treatment Acceptance

Two items, which were adopted from Ortiz, Hull, and Colloca [5], were asked to assess the participants’ acceptance of the placebo treatment: *Do you think it was acceptable for the doctor to try a placebo treatment in this situation?* (item 1, “situational acceptance”) and *If you were the patient, would you be willing to take the treatment described in this way?* (item 2, “personal acceptance”). These items could be answered on five-point Likert scales (“No, not at all,” “No,” “Yes and no,” “Yes,” and “Yes, totally”).

#### Further Measures

In order to investigate baseline differences between the groups regarding their attitude toward medication, the Beliefs about Medicines Questionnaire (BMQ) was used [28]. The questionnaire is divided into two parts: the BMQ-General and the BMQ-Specific. The first assesses beliefs about medication in general and was completed by all participants. It contains 17 items, in total, which provide four subscales: beliefs about the overuse, harm, and benefit of medicines, as well as beliefs about the sensitivity of one’s own body to medicines. The BMQ-Specific assesses beliefs about the participants’ personal medicines that are taken regularly and was therefore only completed by participants with chronic medical conditions. This scale includes two five-item subscales: beliefs about the necessity of the prescribed medicine and concerns about it. All items are rated on five-point Likert scales. There is evidence for both good internal consistency (Cronbach’s *α* = .47–.86) and test-retest reliability (.60–.78), as well as good criterion-related and discriminant validity of the BMQ [28].

To assess the participants’ pre-existing knowledge about placebos and to ensure similar knowledge levels, four questions were asked and a short definition was given, both derived from Fässler, Gnädiger, Rosemann, and Biller-Adorno [3]. Equivalent questions were added about soporifics to ensure that participants were not already expecting a placebo application during the first part of the vignette.

### Statistical Analyses

Statistical analyses were performed using IBM SPSS Statistics 25. The significance level was set at *p* < .05. CEQ scores for each item were z-standardized and averaged to the means for the two factors: credibility and expectancy. To investigate baseline differences between the groups, univariate analyses of variance (ANOVAs) and *χ*^2^ tests were calculated. To investigate within- and between-group differences regarding treatment expectancy and rationale credibility, a 2 × 2 mixed multivariate analysis of variance (MANOVA) was conducted. The MANOVA included the following factors: time (pre-assessment: after case vignette; post-assessment: after treatment vignette), group (DP group; OLP group), and the dependent variables CEQ credibility and CEQ expectancy. Significant main and interaction effects indicated by the overall MANOVA were examined further using univariate ANOVAs and post-hoc *t-*tests (two-tailed). To compare treatment acceptance between the groups, two-tailed *t-*tests were calculated. Within the *t-*tests, the homogeneity of variances was tested via Levene’s tests. When Levene’s tests were significant, the *t-*test statistics were adjusted respectively to correct for potential unequal variances. Bonferroni-corrected significance levels were applied (*p* < .008) to account for possible alpha error cumulation due to multiple comparisons.

For post-hoc analyses (cf. the “Results” section), two separate mediation analyses were conducted using the PROCESS macro for SPSS [32] to investigate whether the effect of group (dummy-coded, OLP = 0, DP = 1) on the two treatment acceptance indices (item 1 “situational acceptance” and item 2 “personal acceptance” as outcome variables) was mediated by outcome expectations (post-assessment CEQ credibility and CEQ expectancy as mediator variables for both analyses).

## Results

### Sample Characteristics

Eight hundred fourteen participants completed the online survey. After the identification and exclusion of outliers (n = 6) and the exclusion of participants with missing data (n = 10), 798 participants were included (DP group: n = 395, OLP group: n = 403). Approximately 76% of the analyzed sample were female, and 71% declared not to suffer from a chronic disease. There were no differences between the groups in demographic variables, health status, beliefs about medicines and placebos, or CEQ scores at pre-assessment (see Tables 1 and 2).

**Table 1:** Sample characteristics at baseline: Demographic variables

	**DP group** (*n* = 395)	**OLP group** (*n* = 403)	**Group differences**
**Age in years**^1^, *n *(%)			*χ*^2^ (4) = 3.00; *p* = .558
18–24	192 (48.6)	175 (43.4)	
25–39	125 (31.6)	145 (36.0)	
40–59	38 (9.6)	45 (11.2)	
60 and older	10 (2.5)	8 (2.0)	
Not specified	30 (7.6)	30 (7.4)	
**Gender**, *n *(%)			*χ*^2^ (2) = 3.02; *p* = .221
Female	300 (75.9)	307 (76.2)	
Male	95 (24.1)	93 (23.1)	
Other	0 (0.0)	3 (0.7)	
**Educational level**^2^, *n *(%)			*χ*^2^ (2) = 3.39; *p* = .183
Below A-level	88 (22.3)	69 (17.1)	
A-level	176 (44.6)	189 (46.9)	
University degree	131 (33.2)	145 (36.0)	
**Health status**^3^, *n *(%)			*χ*^2^ (1) = 0.02; *p* = .895
No chronic disease	282 (71.4)	286 (71.0)	
Chronic disease	113 (28.6)	117 (29.0)	

DP group: Treatment vignette described deceptive placebo application; OLP group: Treatment vignette described open-label placebo application

*M* mean, *SD* standard deviation, *n* number of participants

^1^Age categories are summarized: Answer options were 18–19, 20–24, 25–29, 30–34, 35–39, 40–49, 50–54, 55–59, 60–64, 65, and older

^2^Educational levels are summarized: Answer options were different German school qualifications

^3^Health status categories are summarized: Answer options were healthy, acute illness, mild chronic disease, moderate chronic disease, and severe chronic disease (all explained briefly)

**Table 2:** Sample characteristics at baseline: Beliefs about medicines and placebos

	**DP group**s (*n* = 395)	**OLP group** (*n* = 403)	**Group differences**
**BMQ scores**, *M* (SD)			
General—overuse	3.45 (0.82)	3.41 (0.81)	*F* (1) = 0.37; *p* = .545
General—harm	2.43 (0.74)	2.46 (0.74)	*F* (1) = 0.33; *p* = .566
General—benefit	3.76 (0.75)	3.73 (0.70)	*F* (1) = 0.30; *p* = .586
General—sensitive	2.16 (0.98)	2.19 (0.93)	*F* (1) = 0.17; *p* = .679
Specific—necessity	2.97 (1.17)	2.94 (1.22)	*F* (1) = 0.04; *p* = .842
Specific—concerns	2.03 (0.73)	1.90 (0.68)	*F* (1) = 2.23; *p* = .137
**Attitude placebo**^1^, *n *(%)			*χ*^2^ (3) = 1.60; *p* = .660
Rather positive	100 (25.5)	106 (26.4)	
Rather neutral	248 (63.3)	248 (61.8)	
Rather negative	38 (9.7)	36 (9.0)	
Don’t know	6 (1.5)	11 (2.7)	
**Belief placebo effect**^2^, *n* (%)			*χ*^2^(3) = 3.77; *p* = .287
Often effective	278 (70.4)	272 (67.5)	
Only sometimes effective	108 (27.3)	112 (27.8)	
Not effective	4 (1.0)	10 (2.5)	
Don’t know	5 (1.3)	9 (2.2)	
**CEQ scores,** *M *(SD)			
Credibility	− 0.02 (0.86)	0.02 (0.90)	*F* (1) = 0.28; *p* = .597
Expectancy	0.00 (0.91)	0.00 (0.90)	*F* (1) = 0.01; *p* = .943

DP group: Treatment vignette described deceptive placebo application; OLP group: Treatment vignette described open-label placebo application

*M* mean, *SD* standard deviation, *n* number of participants, *BMQ* Beliefs about Medicines Questionnaire, scores can range between 1 and 5, *CEQ* Credibility/Expectancy Questionnaire, scores are z-standardized

^1^Attitude toward the term “placebo” was measured via the item If you know the term placebo or have ever heard of it: The term is rather positive/neutral/negative for me/I don’t know

^2^Belief in placebo effects was measured via the item Do you think that physical complaints can get better just because you believe in the effect of a therapy? Yes, even quite often/Yes, but only sometimes/No/I don’t know; *CEQ* Credibility/Expectancy Questionnaire, scores are z-standardized

### Rationale Credibility and Treatment Expectancy

A MANOVA indicated a significant main effect of group (*F* [2, 795] = 20.25; *p* ≤ .001) and a significant time × group interaction effect (*F* [2, 795] = 38.97; *p* ≤ .001) using Pillai’s trace. Time did not have a main effect (*F* [2, 795] = 0.01; *p* = .990). Separate univariate ANOVAs also indicated significant effects of group for both credibility (*F* [1, 796] = 31.21; *p* ≤ .001) and expectancy (*F* [1, 796] = 39.05; *p* ≤ .001), as well as significant time × group interaction effects for both credibility (*F* [1, 796] = 65.16; *p* ≤ .001) and expectancy (*F* [1, 796] = 74.35; *p* ≤ .001), meaning that changes in the CEQ scores over time varied between groups.

Bonferroni-corrected post-hoc *t-*tests confirmed that, in both credibility and expectancy, the groups differed significantly at the post-assessment, with higher scores in the DP group than in the OLP group (credibility, corrected after significant Levene test: *t *[791.93] = − 9.42; *p* ≤ .001; expectancy, corrected after significant Levene test: *t* [794.71] = − 10.19; *p* ≤ .001). This indicates that participants’ outcome expectancy and rationale credibility were higher regarding the description of a DP application than regarding the description of an OLP application.

**Fig. 2:**
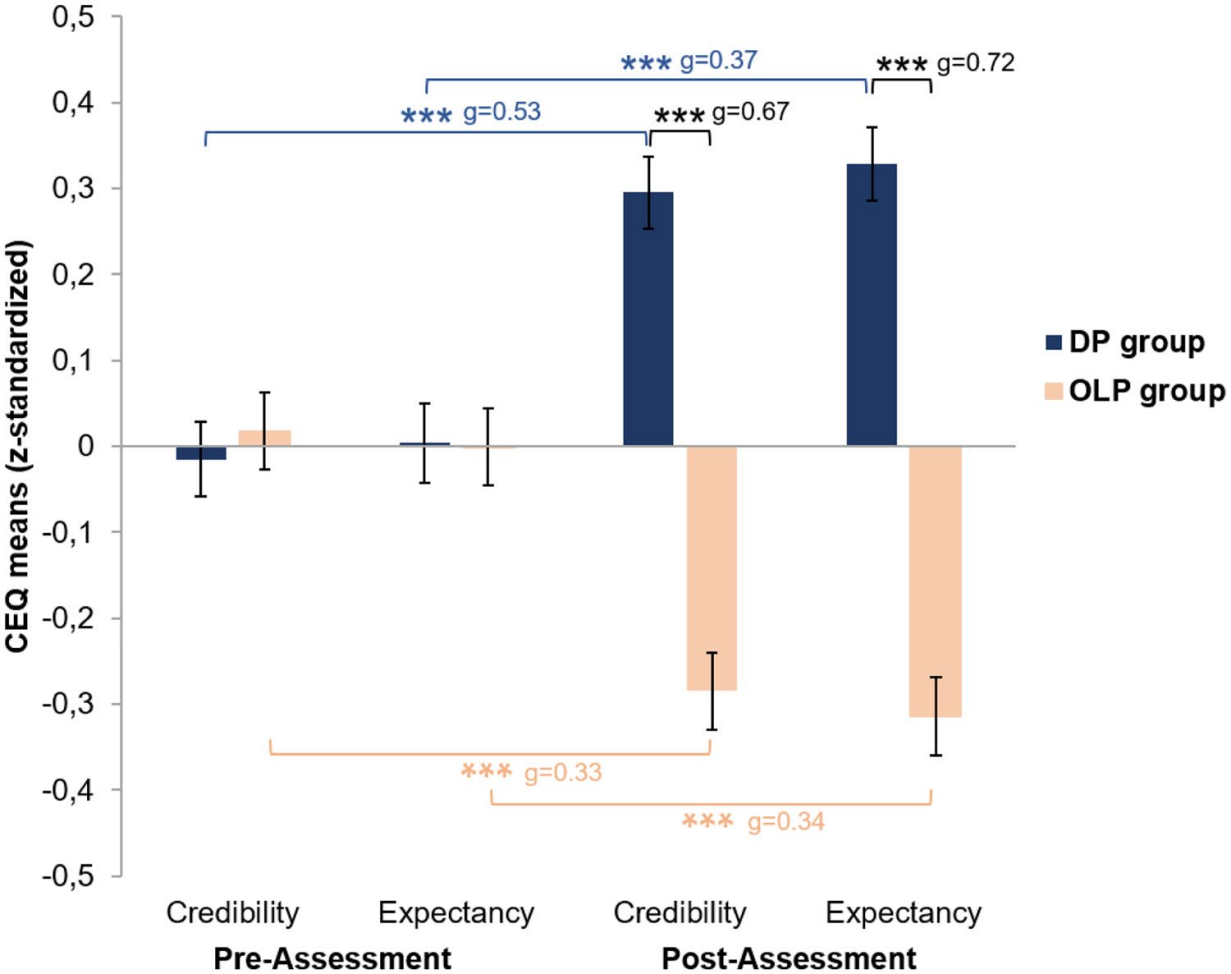
Results of the 2 × 2 mixed multifactorial analysis of variance. CEQ, Credibility/Expectancy Questionnaire. DP group: Treatment vignette described deceptive placebo application; OLP group: Treatment vignette described open-label placebo application. Error bars indicate standard error. Significant effects are indicated by ***p ≤ .001. Bonferroni-corrected threshold: *p* < .008 (all *p* with triple asterisks are significant after Bonferroni correction). Effect sizes: Hedge’s *g*

The Bonferroni-corrected post-hoc *t-*tests confirmed further that, in the DP group, both credibility and expectancy increased from pre- to post-assessment (credibility: *t *[394] = − 7.33; *p* ≤ .001; expectancy: *t* [394] = − 7.62; *p* ≤ .001). However, in the OLP group, both variables decreased (credibility: *t *[402 = 4.83; *p* ≤ .001; expectancy: *t* [402] = 5.21; *p* ≤ .001). These results indicate that participants’ outcome expectancy and rationale credibility were higher after reading the DP treatment vignette than after reading the case vignette. In contrast, when they read the OLP treatment vignette, both outcome expectancy and rationale credibility were lower than they had been after reading the case vignette. CEQ scores for the two groups and two assessment points are shown in Fig. 2.

Due to the high number of participants with chronic diseases in our sample, we replicated the analyses with health status as an additional covariate to control for potential confounding effects. Results are provided in the supplementary material (Tables 4, 5, and 6). All effects as described above remained significant. However, participants without chronic disease showed significantly higher CEQ expectancy scores than participants with chronic disease (*F* [1, 795] = 9.22; *p* = .002).

### Treatment Acceptance

**Fig. 3:**
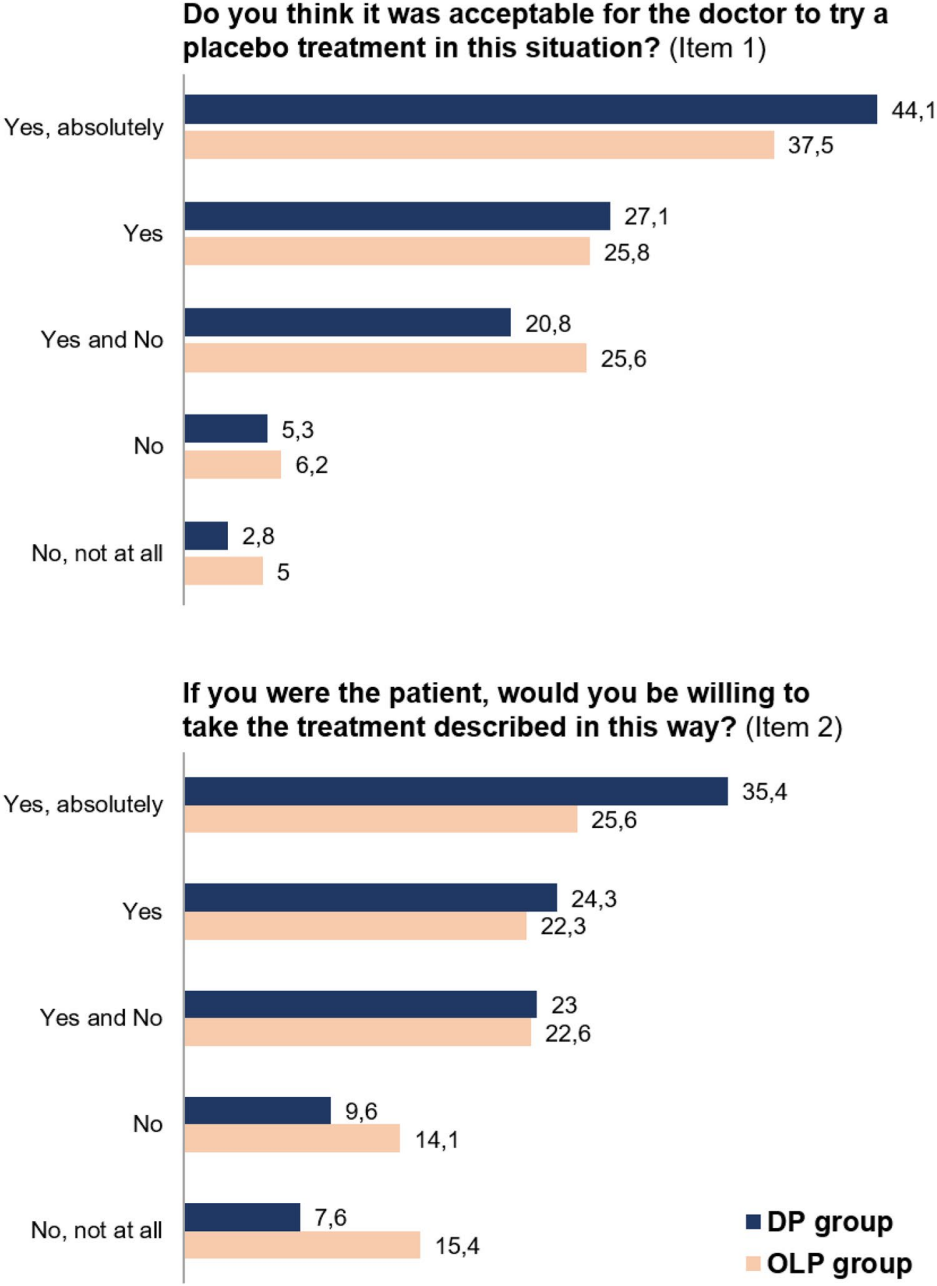
Participants’ ratings of treatment acceptance per group and item in percent. DP group: Treatment vignette described deceptive placebo application. OLP group: Treatment vignette described open-label placebo application

Figure 3 shows the participants’ ratings of treatment acceptance for both groups and both items (see the “Measures” section). In both groups and items, more participants rated the treatment more acceptable than unacceptable. *T-*tests revealed significant differences between the groups with higher scores in the DP group than in the OLP group for both items (item 1 “situational acceptance”: *t* = − 2.51; *p* = .012; item 2 “personal acceptance”: *t* = − 4.47; *p* ≤ .001). This indicates that participants rated the application of a DP as more acceptable than the application of an OLP.

To control for potential confounding effects of chronic diseases, we replicated the treatment acceptance analyses with health status as an additional covariate. The respective results are provided in the supplementary material (Tables 7 and 8). The group effects as described above remained significant. However, participants without chronic disease showed significantly higher personal acceptance than participants with chronic disease (*F* [1, 795] = 7.42; *p* = .007).

### Post-hoc Analyses

**Fig. 4:**
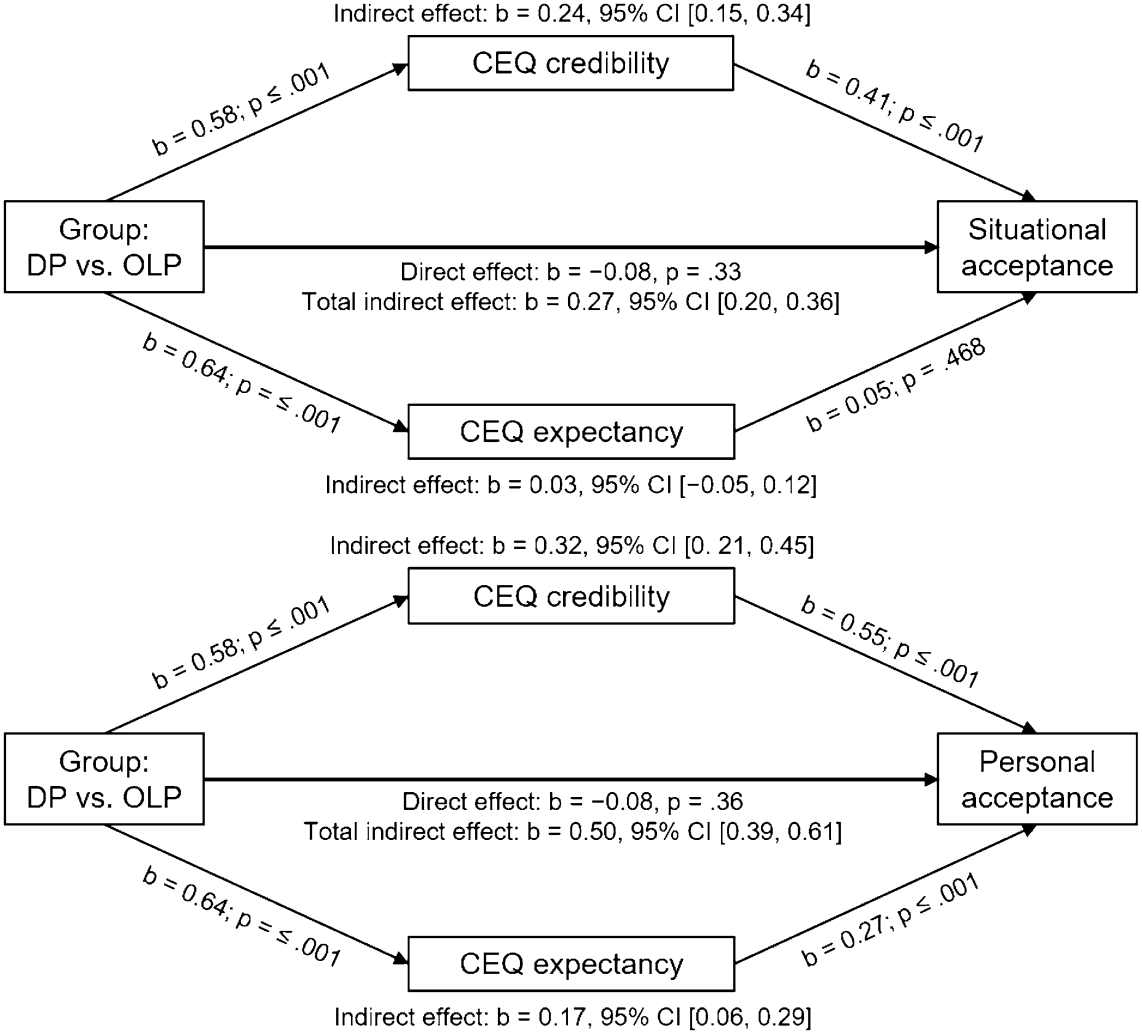
Models of treatment acceptance predicted by group and mediated by rationale credibility and outcome expectancy. The confidence intervals for the indirect effects are bias-corrected and accelerated bootstrapped confidence intervals based on 5000 samples. OLP, open-label placebo; DP, deceptive placebo; CEQ, Credibility/Expectancy Questionnaire

Since the results concerning treatment acceptance were contrary to our hypothesis, we further investigated, via two mediation analyses, whether the effect of group on treatment acceptance was mediated by outcome expectations (CEQ credibility and CEQ expectancy as mediation variables). Diagrams of the mediation models with regression coefficients and direct and indirect effects are shown in Fig. 4.

The mediation with item 1 (“situational acceptance”) as a dependent variable confirmed that there was a significant indirect effect of group on situational acceptance through CEQ credibility (*b* = 0.24; BCa CI [0.15, 0.34]). This indicates that participants reading the DP vignette had higher CEQ credibility scores which, in turn, led to higher situational acceptance. There was no significant indirect effect through CEQ expectancy (*b* = 0.03; BCa CI [− 0.05, 0.12]).

The second post hoc mediation analysis with the same independent and mediating variables but item 2 (“personal acceptance”) as a dependent variable confirmed that there also was a significant indirect effect of group on personal acceptance through CEQ credibility (*b* = 0.32; BCa CI [0.21, 0.45]). Additionally, there was a significant indirect effect through CEQ expectancy (*b* = 0.17; BCa CI [0.06, 0.29]). This indicates that participants reading the DP vignette had higher CEQ credibility and CEQ expectancy scores, which both led to higher personal acceptance.

## Discussion

This experimental online vignette study investigated outcome expectancy, rationale credibility, and treatment acceptance of DPs and OLPs in a large sample of the lay population in Germany. In line with the hypothesis, participants’ outcome expectancy and rationale credibility were higher when they read a vignette about DP treatment than when they read a vignette about OLP treatment. For the DP treatment vignette, outcome expectancy and rationale credibility even increased in comparison with the case vignette read before, which described insomnia and the prescription of unlabeled “pills.” This emphasizes that laypersons’ treatment expectations concerning DPs seem to be high when they read a vignette describing a respective placebo treatment. In contrast, for the OLP treatment vignette, outcome expectancy and rationale credibility decreased compared with the case vignette. These results are in line with qualitative findings of OLP studies, in which participants denied having positive expectations toward the offered OLP treatment [23, 33]. Also, in a qualitative focus group study, most participants suggested that placebos need to be applied with deception to have an effect [7].

Interestingly, our findings contrast with our hypothesis regarding treatment acceptance; participants rated the described DP treatment in insomnia as more acceptable than the described OLP treatment. This effect was the same for situational acceptance, meaning the acceptance of the doctor’s decision in the particular situation, and for personal acceptance, meaning the participants’ personal willingness to take the described treatment in the role of the patient. Since many patients regard physicians’ honesty and transparency in providing information as crucial for medical treatments [4, 5, 7], we expected our participants to rate OLPs, which work without deception, as more acceptable than DPs, which diminish patients’ opportunity to make an autonomous treatment decision. Our finding of DPs being more acceptable also contradicts the results of a previous survey, which found high acceptance scores for OLP treatment [4]. Descriptively, participants in this survey indicated even higher acceptance of OLPs (78.6%) than of DPs (70.9%). However, the differences were not tested for significance. To explore our finding further, we investigated, via post-hoc analyses, whether the effect of higher acceptance in participants reading the DP vignette was mediated by their higher ratings of rationale credibility and outcome expectancy. Regarding situational acceptance, there was an indirect effect through the participants’ ratings of rationale credibility. Regarding personal acceptance, there were indirect effects both through rationale credibility and through outcome expectancy. This might explain our surprising finding. Participants of our study rated the described DP treatment rationale as more credible than the described OLP treatment rationale and (based on the vignettes) believed DPs to be much more effective than OLPs. Situational acceptance was, in turn, predicted by higher rationale credibility, and personal acceptance was predicted by both higher rationale credibility and higher outcome expectancy. These mediation effects could be, at least, one reason why participants rated DPs as more acceptable than OLPs. This interpretation is supported by the mentioned focus group study, in which most participants evaluated placebos from a “consequentialist perspective”; if they expected placebos to be beneficial, they also rated them as acceptable. Thus, participants who did not assume placebos to be effective rated them as unacceptable [7]. Possibly, the participants of our study prioritized potential benefits over the harm to their autonomy by deception. Therefore, they rated that treatment, which they found to be more credible and—when it came to their personal treatment decisions—expected to be more effective, as more acceptable. From this perspective, it is plausible that laypersons do not accept a treatment that they do not expect to improve their symptoms, even if it is explained transparently. Accordingly, our findings regarding outcome expectations and treatment acceptance of different placebo applications still seem to be consistent within our sample. However, since our conclusions are based on a hypothetical vignette scenario and a post-hoc analysis, the proposed explanation needs to be substantiated by further studies.

The large sample size is one of our study’s strengths, contributing to the robustness of our findings. Furthermore, we implemented an innovative online trial design with an experimental character and used well-validated measures. However, some limitations must also be mentioned. First, participants responded to a hypothetical treatment scenario described in a vignette. While the vignette method is a well-established research paradigm and well-suited for experimental designs with high internal validity [34, 35], it cannot fully reflect and mirror real-life treatment experiences. Second, the majority of our sample included young and highly educated females. This limits generalizability. The selectivity of samples, especially concerning gender, is a common problem of online trials or surveys and has been reported in similar studies [4–6]. Third, even though the items we used for the assessment of treatment acceptance have already been used in a previous study by Ortiz et al. [5], it would be preferable to apply scales including several items. Fourth, experimental OLP research must ensure structural equivalence in OLP and control group treatments [36]. We used a very well-established rationale for describing the OLP treatment. However, the wordings of the treatment rationale for OLP and DP might have varied in some aspects that may have inadvertently contributed to differential treatment credibility: Wording of OLP was potentially more technical and referring to scientific results. These aspects contributing to treatment credibility should be subject of further investigations. Finally, we did not integrate a qualitative assessment in our survey, which might have provided additional explanations as to why participants rated OLPs as less acceptable than DPs. Nevertheless, given that qualitative data on this object is already existing, limiting our study to quantitative measures enabled an efficient assessment in a large sample. To the best of our knowledge, our study is one of the very first comparing both outcome expectancy and treatment acceptance of DPs and OLPs in the lay population systematically. Furthermore, the quantitative assessment allowed us to analyze, post-hoc, which variables mediated the effect of group on treatment acceptance, which led to very interesting further results and a plausible explanation for the surprising finding.

Thus, the mentioned limitations can stipulate future research. Replication studies with more heterogeneous demographic characteristics are an important next step. In this context, it could also be interesting to investigate how patient and healthy samples differ in their attitudes concerning placebo applications. This seems to constitute a promising research question, given that our post-hoc covariance analyses indicated that participants with chronic diseases reported lower outcome expectancy and lower personal treatment acceptance as compared with participants without chronic disease. Although health status did not affect our study’s results, additional detailed data on the influence of health status on treatment expectations and acceptance would be important from a clinical point of view. Furthermore, our findings renew questions regarding the role of expectations in OLP treatment. Although positive expectations might not be a major mechanism of OLPs, they seem to be essential to strengthen general acceptance, which, in turn, is crucial if physicians intend to apply OLPs in clinical practice. Some implications for practice can already be inferred. Given that physicians already apply non-specific therapies, i.e., impure placebos, regularly [1, 2], our findings can help indicate when patients might endorse such a treatment: The higher the laypersons’ expectations toward placebos are, the higher the probability that they would find placebo applications acceptable. Therefore, physicians should assess their patients’ expectations before offering a non-specific treatment. There is an ongoing debate as to whether OLPs should be administered in clinical practice. While more research is needed first to establish robust evidence concerning OLP’s effectiveness in specific medical conditions, our study contributes to this debate by providing information on laypersons’ perception of OLPs. Acceptance of OLP seems to rely on positive outcome expectancies. Once OLP’s effectiveness is more clearly established, healthcare professionals would need to assess, discuss, and optimize their patients’ expectancies regarding OLP, before deciding whether to offer a patient OLP treatment. In this instance, OLP treatment could harness positive placebo effects while simultaneously avoiding an unethical deception of the patient.

In conclusion, this study gives important evidence of OLP treatment acceptance and expectancy in the lay population in comparison with DP treatment. It emphasizes that, when laypersons read about placebo treatment, their outcome expectations toward DPs are much higher than toward OLPs and, thus, confirms previous qualitative evidence in an experimental approach with a large layperson sample. While acceptance rates toward placebo treatments, in practice, were generally high, they were even higher toward DPs than toward OLPs. One reason for this might be that higher placebo treatment acceptance was predicted by higher outcome expectations. Our study, therefore, both highlights important directions for future research and contributes to a better understanding of laypersons’ attitudes toward placebo treatments.

## Electronic supplementary material

Below is the link to the electronic supplementary material.

Supplementary file1 (DOCX 13.3 kb)

Supplementary file2 (DOCX 18.1 kb)
