# Supplementary material for: Open-Label Placebo Treatment: Outcome Expectations and General Acceptance in the Lay Population
Source: Int J Behav Med. 2020 Oct 22;28(4):444–54. doi: 10.1007/s12529-020-09933-1 (PMC8263407; doi:10.1007/s12529-020-09933-1)
Supplement: Supplementary file 1 — Supplementary file1 (DOCX 13.3 kb) [file 12529_2020_9933_MOESM1_ESM.docx]

**Supplementary material of the study “Open-label placebo treatment: Outcome expectations and general acceptance in the lay population”**

**Case vignette (all participants)**

Imagine you have suffered from sleep problems for a couple of months. In the evenings you often lie awake for one or two hours. You ruminate about the last day and you toss and turn all the time. Concurrently, you worry about not being well rested the next day and you get annoyed about not falling to sleep.

As a result, you feel tired and exhausted in the daytime. Your family and friends have already asked you about your permanent tiredness.

You make an appointment in a specialized sleep laboratory. In the lab, detailed medical and psychological diagnostics are performed. It appears that you have a sleep disorder which is not due to physical causes or any mental disorder.

The doctor offers you a medication against the sleeping disorder. He gives you a box of pills and says, “I recommend you try these pills. This is a good and effective remedy against sleeping disorders and only has a few side effects. This treatment has helped many of my patients, they feel better now. It is important that you take the pills regularly and carefully, always half an hour before you go to bed with a drink of water.”

**Treatment vignette DP group**

The doctor does not tell you that the pills are in fact placebos without an active agent. You do not even consider the pills to be a fake medication. In the end of the consultation, the doctor gives you the box of pills and you go home. At home, you tell your family of the results of the examination and the doctor’s consultation. In the evening, you take the pill as prescribed by the doctor. You lie down to sleep in the belief that you have taken a sleeping pill with active agent.

**Treatment vignette OLP group**

The doctor explains, “These pills are placebos without an active agent. Scientific research has shown that placebos can have a large positive effect on sleeping disorders and can improve symptoms. Your body has learned that the intake of pills often results in the improvement of symptoms. Therefore, the body can respond automatically to the intake although you know that it is placebo. A positive attitude toward the treatment can augment this effect but is not necessary. So you do not have to believe in this to benefit from the placebo.”
