# Supplementary material for: Open-Label Placebo Treatment: Outcome Expectations and General Acceptance in the Lay Population
Source: Int J Behav Med. 2020 Oct 22;28(4):444–54. doi: 10.1007/s12529-020-09933-1 (PMC8263407; doi:10.1007/s12529-020-09933-1)
Supplement: Supplementary file 2 — Supplementary file2 (DOCX 18.1 kb) [file 12529_2020_9933_MOESM2_ESM.docx]

**Supplementary material of the study “Open-label placebo treatment: Outcome expectations and general acceptance in the lay population”**

| Table 3  *Means and standard deviations of z-standardized CEQ scores within both groups and at both assessment points.* | | |
| --- | --- | --- |
|  | **Credibility,**  ***Mean (SD)*** | **Expectancy,**  ***Mean (SD)*** |
| **Deceptive placebo group**  Pre-Assessment  Post-Assessment | −0.02 (0.86)  0.30 (0.83) | 0.00 (0.91)  0.33 (0.86) |
| **Open-label placebo group**  Pre-Assessment  Post-Assessment | 0.02 (0.90)  −0.28 (0.91) | 0.00 (0.90)  −0.31 (0.92) |
| *Note:* Deceptive placebo group: Treatment vignette described deceptive placebo application; Open-label placebo group: Treatment vignette described open-label placebo application; CEQ = Credibility/Expectancy Questionnaire. | | |

| Table 4  *Means and standard deviations of z-standardized CEQ scores in participants with and without chronic disease at both assessment points.* | | |
| --- | --- | --- |
|  | **Credibility,**  ***Mean (SD)*** | **Expectancy,**  ***Mean (SD)*** |
| **Participants without chronic disease**  Pre-Assessment  Post-Assessment | 0.02 (0.86)  0.02 (0.91) | 0.05 (0.88)  0.06 (0.95) |
| **Participants with chronic disease**  Pre-Assessment  Post-Assessment | −0.05 (0.91)  −0.05 (0.94) | −0.11 (0.94)  −0.14 (0.93) |
| *Note:* CEQ = Credibility/Expectancy Questionnaire. | | |

| Table 5  *Main and interaction effects of a 2x2 mixed multivariate analysis of covariance, including time (pre-assessment; post-assessment) and group (DP group; OLP group) as factor variables, health status (chronic disease; no chronic disease) as covariate, and CEQ credibility and CEQ expectancy as dependent variables.* | | | |
| --- | --- | --- | --- |
|  | ***F*** | ***df*** | ***p*** |
| **Between subjects**  Group  Health status | 20.32  5.94 | 2, 794  2, 794 | ≤ .001  .003 |
| **Within subjects**  Time  Time x group  Time x health status | 0.52  38.91  0.56 | 2, 794  2, 794  2, 794 | .592  ≤ .001  .573 |
| *Note:* Test statistic is Pillai’s trace; DP group: Treatment vignette described deceptive placebo application; OLP group: Treatment vignette described open-label placebo application; CEQ = Credibility/Expectancy Questionnaire. | | | |

| Table 6  *Main and interaction effects of 2x2 mixed analyses of covariance following the overall multivariate analysis of covariance, including time (pre-assessment; post-assessment) and group (DP group; OLP group) as factor variables, health status (chronic disease; no chronic disease) as covariate, and CEQ credibility and CEQ expectancy as dependent variables.* | | | |
| --- | --- | --- | --- |
|  | ***F*** | ***df*** | ***p*** |
| **Credibility**  Time  Group  Health status  Time x group  Time x health status | 0.01  31.16  1.66  65.08  0.01 | 1, 795  1, 795  1, 795  1, 795  1, 795 | .944  ≤ .001  .198  ≤ .001  .911 |
| **Expectancy**  Time  Group  Health status  Time x group  Time x health status | 0.30  39.27  9.22  74.23  0.29 | 1, 795  1, 795  1, 795  1, 795  1, 795 | .582  ≤ .001  .002  ≤ .001  .593 |
| *Note:* DP group: Treatment vignette described deceptive placebo application; OLP group: Treatment vignette described open-label placebo application; CEQ = Credibility/Expectancy Questionnaire. | | | |

| Table 7  *Means and standard deviations of treatment acceptance scores in participants with and without chronic disease.* | | |
| --- | --- | --- |
|  | **Situational acceptance,**  ***Mean (SD)*** | **Personal acceptance,**  ***Mean (SD)*** |
| **Participants without chronic disease** | 3.98 (1.10) | 3.57 (1.32) |
| **Participants with chronic disease** | 3.85 (1.14) | 3.29 (1.38) |
| *Note:* Situational acceptance was measured via item 1: “Do you think it was acceptable for the doctor to try a placebo treatment in this situation?”; Personal acceptance was measured via item 2: “If you were the patient, would you be willing to take the treatment described in this way?”. | | |

| Table 8  *Results of univariate analyses of covariance, including group (DP group; OLP group) as factor variable, health status (chronic disease; no chronic disease) as covariate, and both acceptance items as dependent variables.* | | | |
| --- | --- | --- | --- |
|  | ***F*** | ***df*** | ***p*** |
| **Situational acceptance**  Group  Health status | 6.37  2.20 | 1, 795  1, 795 | .012  .138 |
| **Personal acceptance**  Group  Health status | 20.01  7.42 | 1, 795  1, 795 | ≤ .001  .007 |
| *Note:* DP group: Treatment vignette described deceptive placebo application; OLP group: Treatment vignette described open-label placebo application; Situational acceptance was measured via item 1: “Do you think it was acceptable for the doctor to try a placebo treatment in this situation?”; Personal acceptance was measured via item 2: “If you were the patient, would you be willing to take the treatment described in this way?”. | | | |
